# Supplementary material for: Conditional cash transfer and mortality among interpersonal violence victims: A cohort study
Source: PLoS Med. 2026 May 6;23(5):e1004673. doi: 10.1371/journal.pmed.1004673 (PMC13148827; doi:10.1371/journal.pmed.1004673)
Supplement: S1 Appendix — Method A – Description of the main datasets used in the study. Method B – Description of the linkage. Table A – Logistic regression to estimate propensity scores for receiving Bolsa Familia Program according to covariates. Table B – Association of Bolsa Família Program participation with overall mortality by sex (2011–2015). Table C – Association of Bolsa Família Program participation with the outcomes using Poisson model. Table D – Association of Bolsa Família Program participation with the outcomes considering overall population who were victim of interpersonal violence (2011–2015). Table E – Association of Bolsa Família Program participation with overall mortality considering missing as a category (2011–2015). Table F – Crude association of Bolsa Família Program participation with overall mortality (2011–2015). Table G – Association of Bolsa Família Program participation with overall mortality using time-varying BFP status (2011–2015). Table H – Information Criteria–Based Model Comparison Evaluating the Inclusion of an Interaction Term. Table I – Association of Bolsa Família Program participation with overall mortality considering other age categorization in the propensity score estimation (2011–2015). Fig A – Distribution of the propensity score in the sample, 2011–2015. (DOCX) [file pmed.1004673.s001.docx]

**Appendix**

**Conditional cash transfer and mortality among interpersonal violence victims: a cohort study**

**Method A - Description of the main datasets used in the study**

**100 million Brazilian Cohort baseline**

The 100 million Brazilian Cohort baseline is a dataset developed by Center for Data and Knowledge Integration for Health (CIDACS / FIOCRUZ) to investigate social determinants and the impact of social programs and policies on various health contexts in Brazil. This dataset is based on information from over 131 million individuals who were registered between 2001 and 2018 in CadÚnico, the primary system for applying for social benefits in Brazil which include the Bolsa Família Program (BFP)^3,5^. It includes socio-economic and sociodemographic information from poorer Brazilian individuals and their families who apply for social programs such as BFP^5^. To qualify and register with CadÚnico, families must have a per capita income of up to half a minimum wage or a total family income of up to three minimum wages^5^.

**Hospitalization Information System (SIH)**

The SIH is the national dataset for all hospitalizations funded by the Brazilian public health system, including both general and specialized hospitals^6^. Although Brazil also has a private health system, around 75% of the Brazilian population relies on the public system^7^. The SIH was filled out by health professionals who use standardised forms according to updated International Classification of Disease (ICD-10) to record primary and secondary causes of a hospital admission^7^. The record of interpersonal violence admissions uses codes X85-Y09 from the International Classification of Diseases (ICD-10)^8^ for aggression, which are recorded as secondary diagnoses, as well as other external causes^9^. However, to capture all possible hospitalizations related to this cause within the system, we also include records where it appears as the primary diagnosis.

**Mortality Information System (SIM)**

The SIM is a dataset responsible for the mandatory registration of all deaths in Brazil^6^. This system is also filled out by health professionals using standardized forms based on the updated ICD-10 for all causes of death^6^. The SIM has been recognized for its high quality and completeness^10^.

**National Disease Notification System (SINAN)**

Brazil has had a compulsory system for recording interpersonal violence since 2011^11^, that was established by the National Policy for Reducing Morbidity and Mortality from Accidents and Violence from the Brazilian government^11^, and it has improved its reporting over the years^12^. The data are registered by professionals working in health facilities as well as in other sectors, such as public security and law enforcement, social assistance services, educational sector and judicial system^11^. However, healthcare services remain the primary source of reporting in SINAN, as they are responsible for mandatory notifications of violence-related injuries, infectious diseases, and other health conditions^11^. All records are uploaded to the National Disease Notification System (SINAN), encompassing self-harm and interpersonal violence, including physical, sexual, psychological abuse, and neglect, as well as the type of relationship with the perpetrator^11^. For this study, we excluded self-harm records and cases where the perpetrator was the own individual.

**Method B - Description of the linkage**

The data from the 100 million Brazilian Cohort baseline was linked with records of the Bolsa Família Program (BFP) payments, the Hospital Information System (SIH), the Mortality Information System (SIM) and the National Disease Notification System (SINAN). The linkage between the cohort and BFP utilized a deterministic approach, relying on a common correspondence key between the two databases (social identification number). Subsequently, record linkage using CIDACS-RL^1,2^, a tool for linking individual records in two stages using identifiers, was employed to connect information from the cohort baseline (2001-2018) with SIH (2008-2018), SIM (2000-2015) and SINAN (2009-2018). This process involved utilizing variables such as the name, mother's name, date of birth, sex, and municipality of residence^1,2^. The initial stage comprised deterministic linkage of five variables, followed by the second stage based on a similarity index derived from these variables^2^. To assess the accuracy of the linkage, a manual verification of a randomly selected sample was performed and evaluated through a receiver operating characteristic curve, considering sensitivity and specificity indexes. All linkage procedures were executed at the Center for Data and Knowledge Integration for Health (CIDACS)/ Fiocruz^3^, within a stringent data protection environment and in adherence to ethical and legal standards^4^. The results indicated that the linkage demonstrated accuracy and sensitivity over 90%.

**eReferences**

1 Almeida D, Gorender D, Ichihara MY, Sena S, Menezes L, Barbosa GCG, et al. Examining the quality of record linkage process using nationwide Brazilian administrative databases to build a large birth cohort. *BMC Med Inform Decis Mak.* 2020; 20:173.

2 Barbosa GCG, Ali MS, Araujo B, Reis S, Sena S, Ichihara MYT, et al. CIDACS-RL: a novel indexing search and scoring-based record linkage system for huge datasets with high accuracy and scalability. *BMC Med Inform Decis Mak*. 2020;20:289.

3 Barreto ML, Ichihara MY, Almeida BA, Barreto ME, Cabral L, Fiaccone RL, et al. The Center for Data and Knowledge Integration for Health (CIDACS): Linking health and social data in Brazil. *Int J Popul Data Sci*. 2019; 4:1-12.

4 Harron K, Dibben C, Boyd J, Hjern A, Azimaee M, Barreto ML, et al. Challenges in administrative data linkage for research. *Big Data Soc.* 2017; 4:2053951717745678.

5 Barreto ML, Ichiara MY, Pescarini JM, Ali MS, Borges GL, Fiaccone RL, et al. Cohort Profile: The 100 Million Brazilian Cohort. *Int J Epidemiol*. 2022; 51: e27-e38.

6 Machado DB, Azevedo JPA, Alves FJO, Castro-de-Araujo LFS, Silva ER, Fialho EMX, et al. The impact of social drivers, conditional cash transfers and their mechanisms on the mental health of the young; an integrated retrospective and forecasting approach using the 100 million Brazilian Cohort: A study protocol*. PLoS One*. 2022; 17: e0272481.

7 Cerqueira DRC, Alves PP, Coelho DCS, Reis MVM, Lima AS. Uma análise da base de dados do Sistema de Informação Hospitalar entre 2001 e 2018: dicionário dinâmico, disponibilidade dos dados e aspectos metodológicos para a produção de indicadores sobre violência. Rio de Janeiro: IPEA, 2019.

8 World Health Organization (WHO) ICD-10: international statistical classification of diseases and related health problems: tenth revision.2nd ed. Geneva: World Health Organization, 2004.

9 Brasil. Ministério da Saúde. Morbidade Hospitalar do SUS por Causas Externas por local de internação - Notas Técnicas. <http://tabnet.datasus.gov.br/cgi/sih/eidescr.htm#descvar>. (accessed Mar 11, 2025).

10 World Health Organization (WHO). WHO methods and data sources for country-level causes of death 2000-2019. Geneva: WHO, 2020.

11 Brasil. Ministério da Saúde. Secretaria de Vigilância em Saúde. Departamento de Vigilância de Doenças e Agravos Não Transmissíveis e Promoção da Saúde. Viva: instrutivo notificação de violência interpessoal e autoprovocada. 2. ed. Brasília: Ministério da Saúde, 2016.

12 Sousa CM de S, Mascarenhas MDM, Lima PVC, Rodrigues MTP. Incompletude do preenchimento das notificações compulsórias de violência - Brasil, 2011-2014. *Cad saúde colet*. 2020; 28:477–87.

**Table A - Logistic regression to estimate propensity scores for receiving Bolsa Familia Program according to covariates, N=26,087**

| **Variable** | **Odds Ratio**  **(95% CI)** | **p value** |
| --- | --- | --- |
| Sex  Male  Female | 1.00  1.55 (1.46, 1.64) | <0.001 |
| Age group (years old)  <10  10-24  25-59  >60 | 1.00  0.64 (0.57, 0.71)  0.47 (0.42, 0.51)  0.07 (0.06, 0.09) | <0.001  <0.001  <0.001 |
| Education Level (years of education)  Never been study  Preschool  Primary school or less (<= 5 years)  Junior high school (6- 10 years)  High school (10-12 years)  College/university (>=13 years) | 1.00  0.78 (0.69, 0.88)  0.85 (0.77, 0.93)  0.95 (0.85, 1.07)  0.74 (0.66, 0.83)  0.30 (0.23, 0.38) | <0.001  <0.001  0.398  <0.001  <0.001 |
| Race  White  Black  Asian  Brown  Indigenous | 1.00  1.16 (0.98, 1.38)  1.54 (1.05, 2.25)  0.96 (0.84, 1.10)  1.61 (0.98, 2.66) | 0.082  0.027  0.616  0.061 |
| Location of residence  Urban  Rural | 1.00  0.91 (0.79, 1.04) | 0.157 |
| Brazilian regions  Southeast  Northeast  Midwest  South  North | 1.00  0.98 (0.90, 1.07)  0.60 (0.54, 0.65)  0.64 (0.59, 0.69)  0.82 (0.73, 0.92) | 0.631  <0.001  <0.001  <0.001 |
| Household characteristics  Water supply  Public Network (running water)  Well, natural sources, or other  Waste  Public collection system  Burned, buried, outdoor disposal, o other  Sanitation  Public network  Septic tank  Homemade septic tank  Ditch or other  Construction materials  Bricks/ cement  Wood, other vegetal materials, and other | 1.00  1.30 (1.18, 1.43)  1.00  0.93 (0.80, 1.09)  1.00  0.98 (0.90, 1.06)  1.01 (0.93, 1.09)  1.27 (1.08, 1.49)  1.00  1.39 (1.27, 1.52) | <0.001  0.391  0.594  0.919  0.004  0.391 |
| Isolation  Live with someone else  Live alone | 1.00  0.71 (0.64, 0.78) | <0.001 |
| Year of registration at CadÚnico  2011  2012  2013  2014  2015 | 1.00  0.80 (0.68, 0.94)  0.60 (0.51, 0.70)  0.40 (0.34, 0.47)  0.24 (0.21, 0.28) | 0.006  <0.001  <0.001  <0.001 |

Abbreviation: CI, Confidence Interval.

**Table B - Association of Bolsa Família Program participation with overall mortality by sex (2011-2015).**

|  | **Cox model** | **Competing risk model** | |
| --- | --- | --- | --- |
| **Confounder adjustment** | **Overall mortality** | **Natural causes** | **Unnatural causes** |
|  | **HR^1^ (95%CI)** | **HR^1^ (95%CI)** | **HR^1^ (95%CI)** |
| Sex  Male  N  p value  Female  N  p value | 0.90 (0.74 – 1.12)  9,876  0.360  0.42 (0.34 – 0.52)  16,217  <0.001 | 0.69 (0.52 – 0.91)  9,876  0.009  0.61 (0.46 – 0.80)  16,217  0.001 | 1.40 (1.00 – 1.94)  9,876  0.045  1.82 (0.98 – 3.35)  16,217  0.006 |

^1^ HR estimated with stabilized IPTW given for sex, age, race, educational level, household conditions (water supply, waste, sanitation, construction materials), living alone, Brazilian region, location of residence, year of hospitalization or occurrence of interpersonal violence, and year of registration at CadÚnico.

Abbreviations: HR, Hazard Ratio; CI, Confidence Interval.

**Table C - Association of Bolsa Família Program participation with the outcomes using Poisson model.**

|  |  | **Overall mortality** | **Natural causes** | **Unnatural causes** |
| --- | --- | --- | --- | --- |
| **Confounder adjustment** | **Overall population** | **IRR (95% CI)** | **IRR (95% CI)** | **IRR (95% CI)** |
| Poisson adjusted with stabilized IPTW^1^  Non-BFP  BFP  p value | 26,093 | 1.00  0.50 (0.43, 0.59)  <0.001 | 1.00  0.37 (0.31, 0.45)  <0.001 | 1.00  0.90 (0.68, 1.20)  0.489 |

^1^ IRR estimated with stabilized IPTW given for sex, age, race, educational level, household conditions (water supply, waste, sanitation, construction materials), living alone, Brazilian region, location of residence, year of hospitalization or occurrence of interpersonal violence, and year of registration at CadÚnico.

Abbreviations: BFP, Bolsa Família Program; IRR, Incidence Rate Ratio; CI, Confidence Interval; IPTW, Inverse Propensity Scores.

**Table D - Association of Bolsa Família Program participation with the outcomes considering overall population who were victim of interpersonal violence (2011-2015), N= 64,066**

|  |  | **Cox model** | **Competing risk model** | |  |
| --- | --- | --- | --- | --- | --- |
|  |  | **Overall mortality**  **N= 2,779** | **Natural causes**  **N= 1,736** | **Unnatural causes**  **N= 1,043** | |
| **Confounder adjustment** | **Overall population** | **HR (95%CI)** | **HR (95%CI)** | **HR (95%CI)** | |
| Cox adjusted with stabilized IPTW^1^  Non-BFP  BFP  p value | 64,066 | 1.00  0.83  (0.75, 0.93)  <0.001 | 1.00  0.73  (0.63, 0.83)  <0.001 | 1.00  1.05  (0.90, 1.22)  0.513 | |

^1^ HR estimated with stabilized IPTW given for sex, age, race, educational level, household conditions (water supply, waste, sanitation, construction materials), living alone, Brazilian region, location of residence, year of hospitalization or occurrence of interpersonal violence, and year of registration at CadÚnico.

Abbreviations: BFP, Bolsa Família Program; HR, Hazard Ratio; CI, Confidence Interval; IPTW, Inverse Propensity Scores.

**Table E - Association of Bolsa Família Program participation with overall mortality considering missing as a category (2011-2015).**

|  |  | **Cox model** | **Competing risk model** | |  |
| --- | --- | --- | --- | --- | --- |
|  |  | **Overall mortality**  **N= 2,779** | **Natural causes**  **N= 1,736** | **Unnatural causes**  **N= 1,043** | |
| **Confounder adjustment** | **Overall population** | **HR (95%CI)** | **HR (95%CI)** | **HR (95%CI)** | |
| Cox adjusted with stabilized IPTW^1^  Non-BFP  BFP  p value | 29,075 | 1.00  0.89  (0.77, 0.99)  0.050 | 1.00  0.74  (0.62, 0.89)  0.001 | 1.00  1.31  (1.01, 1.70)  0.045 | |

^1^ HR estimated with stabilized IPTW given for sex, age, race, educational level, household conditions (water supply, waste, sanitation, construction materials), living alone, Brazilian region, location of residence, year of hospitalization or occurrence of interpersonal violence, and year of registration at CadÚnico.

Abbreviations: BFP, Bolsa Família Program; HR, Hazard Ratio; CI, Confidence Interval; IPTW, Inverse Propensity Scores.

**Table F – Crude association of Bolsa Família Program participation with overall mortality (2011-2015).**

| **Confounder adjustment** | **Overall population** | **HR (95%CI)** |
| --- | --- | --- |
| Cox  Non-BFP  BFP  p value | 29,075 | 1.00  0.47 (0.41 – 0.53)  <0.001 |

Abbreviations: BFP, Bolsa Família Program; HR, Hazard Ratio; CI, Confidence Interval; IPTW.

**Table G - Association of Bolsa Família Program participation with overall mortality using time-varying BFP status (2011-2015).**

| **Confounder adjustment** | **Overall population** | **HR (95%CI)** |
| --- | --- | --- |
| Cox adjusted with stabilized IPTW^1^  Non-BFP  BFP  p value | 26,093 | 1.00  0.70  (0.58, 0.83)  <0.001 |

^1^ HR estimated with stabilized IPTW given for sex, age, race, educational level, household conditions (water supply, waste, sanitation, construction materials), living alone, Brazilian region, location of residence, year of hospitalization or occurrence of interpersonal violence, and year of registration at CadÚnico.

Abbreviations: BFP, Bolsa Família Program; HR, Hazard Ratio; CI, Confidence Interval; IPTW, Inverse Propensity Scores.

**Table H – Information Criteria–Based Model Comparison Evaluating the Inclusion of an Interaction Term.**

| **Models** | **Observations** | **Log-likelihood** | **Degrees of freedom** | **AIC** | **BIC** |
| --- | --- | --- | --- | --- | --- |
| Model 1 (main effect) | 26,093 | -6997.589 | 5 | 14005.18 | 14046.02 |
| Model 2 (main effect + interaction term) | 26,093 | -6579.473 | 11 | 13180.95 | 13270.81 |

Abbreviations: AIC, Akaike Information Criterion; BIC, Bayesian Information Criterion

**Table I - Association of Bolsa Família Program participation with overall mortality considering other age categorization in the propensity score estimation (2011-2015).**

|  |  | **Cox model** | **Competing risk model** | |  |
| --- | --- | --- | --- | --- | --- |
|  |  | **Overall mortality**  **N= 2,779** | **Natural causes**  **N= 1,736** | **Unnatural causes**  **N= 1,043** | |
| **Confounder adjustment** | **Overall population** | **HR (95%CI)** | **HR (95%CI)** | **HR (95%CI)** | |
| Cox adjusted with stabilized IPTW^1^  Non-BFP  BFP  p value | 26,093 | 1.00  0.87  (0.74, 1.02)  0.094 | 1.00  0.73  (0.60, 0.89)  0.002 | 1.00  1.33  (0.99, 1.77)  0.053 | |

^1^ HR estimated with stabilized IPTW given for sex, age (categories: <10 years, 10–19 years, 20–29 years, 30–39 years, 40-49 years, 50–59 years, and >60 years), race, educational level, household conditions (water supply, waste, sanitation, construction materials), living alone, Brazilian region, location of residence, year of hospitalization or occurrence of interpersonal.

Abbreviations: BFP, Bolsa Família Program; HR, Hazard Ratio; CI, Confidence Interval; IPTW, Inverse Propensity Scores.

**Fig A- Distribution of the propensity score in the sample, 2011-2015**


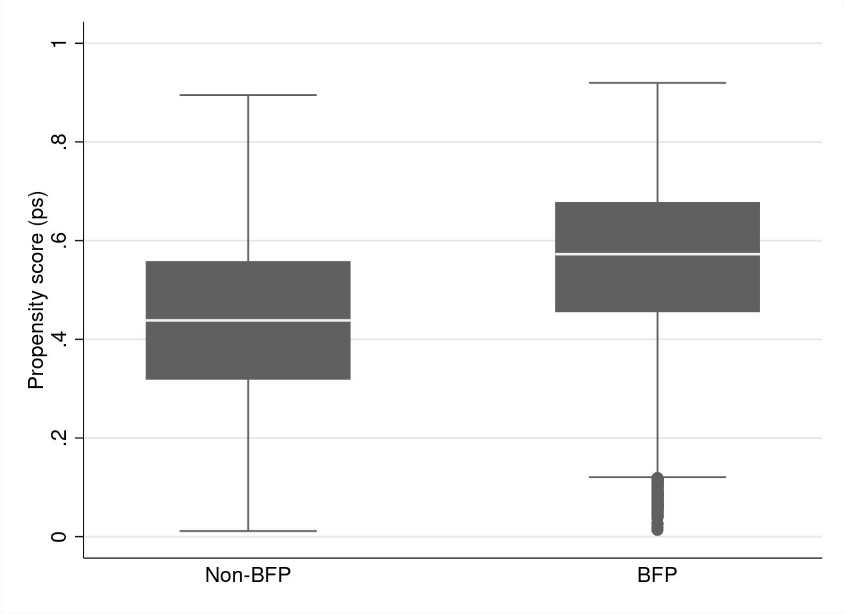


Abbreviations: BFP, Bolsa Familia Program; ps, propensity score
