# Supplementary material for: Conditional cash transfer and mortality among interpersonal violence victims: A cohort study
Source: PLoS Med. 2026 May 6;23(5):e1004673. doi: 10.1371/journal.pmed.1004673 (PMC13148827; doi:10.1371/journal.pmed.1004673)
Supplement: S1 STROBE Checklist — Information on the STROBE Initiative is available at www.strobe-statement.org. (DOC) [file pmed.1004673.s002.doc]

**STROBE Statement—checklist of items that should be included in reports of observational studies**

|  | Item No | Recommendation | Location in the manuscript where items are reported |
| --- | --- | --- | --- |
| **Title and abstract** | 1 | (*a*) Indicate the study’s design with a commonly used term in the title or the abstract | Page 1 |
|  |  | (*b*) Provide in the abstract an informative and balanced summary of what was done and what was found | Page 2 |
| Introduction | | |  |
| Background/rationale | 2 | Explain the scientific background and rationale for the investigation being reported | Paragraphs 3, 4, 5 in the Introduction |
| Objectives | 3 | State specific objectives, including any prespecified hypotheses | Last Paragraph in the Introduction section |
| Methods | | |  |
| Study design | 4 | Present key elements of study design early in the paper | Paragraph 1 in the Methods section |
| Setting | 5 | Describe the setting, locations, and relevant dates, including periods of recruitment, exposure, follow-up, and data collection | Paragraphs 1, 2, 3, 4 in the Methods section, “Follow-up section”, “Exposure” section |
| Participants | 6 | (*a*) *Cohort study*—Give the eligibility criteria, and the sources and methods of selection of participants. Describe methods of follow-up  *Case-control study*—Give the eligibility criteria, and the sources and methods of case ascertainment and control selection. Give the rationale for the choice of cases and controls  *Cross-sectional study*—Give the eligibility criteria, and the sources and methods of selection of participants | Participants section |
|  |  | (*b*) *Cohort study*—For matched studies, give matching criteria and number of exposed and unexposed  *Case-control study*—For matched studies, give matching criteria and the number of controls per case | Paragraphs 2 and 3 in the Statistical analyses section |
| Variables | 7 | Clearly define all outcomes, exposures, predictors, potential confounders, and effect modifiers. Give diagnostic criteria, if applicable | Exposure section, and Outcomes section |
| Data sources/ measurement | 8* | For each variable of interest, give sources of data and details of methods of assessment (measurement). Describe comparability of assessment methods if there is more than one group | Exposure and Outcome sections, Paragraphs 2 and 3 in the Statistical analyses section |
| Bias | 9 | Describe any efforts to address potential sources of bias | Paragraphs 2 and 3 in the Statistical Analyses section |
| Study size | 10 | Explain how the study size was arrived at | Paragraph 1 and 2 in the Study design section |
| Quantitative variables | 11 | Explain how quantitative variables were handled in the analyses. If applicable, describe which groupings were chosen and why | Exposure and Outcome sections |
| Statistical methods | 12 | (*a*) Describe all statistical methods, including those used to control for confounding | Paragraphs 1 to 6 of Statistical Analyses section |
|  |  | (*b*) Describe any methods used to examine subgroups and interactions | Paragraphs 1 in Sensitivity analyses section |
|  |  | (*c*) Explain how missing data were addressed | Paragraphs 1 in Sensitivity analyses section |
|  |  | (*d*) *Cohort study*—If applicable, explain how loss to follow-up was addressed  *Case-control study*—If applicable, explain how matching of cases and controls was addressed  *Cross-sectional study*—If applicable, describe analytical methods taking account of sampling strategy | Paragraph 2 of Participant section |
|  |  | (*e*) Describe any sensitivity analyses | Paragraphs 1 in Sensitivity analyses section |

Continued on next page

| Results | | |  |  |
| --- | --- | --- | --- | --- |
| Participants | 13* | (a) Report numbers of individuals at each stage of study—eg numbers potentially eligible, examined for eligibility, confirmed eligible, included in the study, completing follow-up, and analysed | Paragraph 1 in the Results section |  |
|  |  | (b) Give reasons for non-participation at each stage | Paragraph 1 in the Results section |  |
|  |  | (c) Consider use of a flow diagram | Figure 1 |  |
| Descriptive data | 14* | (a) Give characteristics of study participants (eg demographic, clinical, social) and information on exposures and potential confounders | Paragraphs 1 and in the Results section |  |
|  |  | (b) Indicate number of participants with missing data for each variable of interest |  |  |
|  |  | (c) *Cohort study*—Summarise follow-up time (eg, average and total amount) |  |  |
| Outcome data | 15* | *Cohort study*—Report numbers of outcome events or summary measures over time | Paragraph 3 in the Results section | |
|  |  | *Case-control study—*Report numbers in each exposure category, or summary measures of exposure |  |  |
|  |  | *Cross-sectional study—*Report numbers of outcome events or summary measures |  |  |
| Main results | 16 | (*a*) Give unadjusted estimates and, if applicable, confounder-adjusted estimates and their precision (eg, 95% confidence interval). Make clear which confounders were adjusted for and why they were included | Paragraph 4 in the Results section |  |
|  |  | (*b*) Report category boundaries when continuous variables were categorized |  |  |
|  |  | (*c*) If relevant, consider translating estimates of relative risk into absolute risk for a meaningful time period |  |  |
| Other analyses | 17 | Report other analyses done—eg analyses of subgroups and interactions, and sensitivity analyses | Paragraph 5 and 6 in the Results section |  |
| Discussion | | |  |  |
| Key results | 18 | Summarise key results with reference to study objectives | Paragraph 1 in the Discussion section |  |
| Limitations | 19 | Discuss limitations of the study, taking into account sources of potential bias or imprecision. Discuss both direction and magnitude of any potential bias | Paragraphs 8 to 11 in the Discussion section |  |
| Interpretation | 20 | Give a cautious overall interpretation of results considering objectives, limitations, multiplicity of analyses, results from similar studies, and other relevant evidence | Paragraphs 2 to 7 in the Discussion section |  |
| Generalisability | 21 | Discuss the generalisability (external validity) of the study results | Paragraph 13 in the Discussion section |  |
| Other information | | |  |  |
| Funding | 22 | Give the source of funding and the role of the funders for the present study and, if applicable, for the original study on which the present article is based | Role of the funding section in the Funding Statement section |  |
