# Supplementary material for: Conditional cash transfer and mortality among interpersonal violence victims: A cohort study
Source: PLoS Med. 2026 May 6;23(5):e1004673. doi: 10.1371/journal.pmed.1004673 (PMC13148827; doi:10.1371/journal.pmed.1004673)
Supplement: Attachment — Submitted filename: Point by Point_PlosMedicine v4.docx [file pmed.1004673.s003.docx]

Dear Dr. Syba Sunny,

Thank you for giving us the opportunity to resubmit a revised version of our manuscript. We greatly appreciate the time and effort that you and the reviewers have put into providing valuable feedback on our work.

We have incorporated most of suggestions provided and present a manuscript free of inconsistencies, as well as made a few insertions and English corretions to clarify some information.

Below is a point-by-point response to the editors' and reviewers' comments and requests.

Please note that the line numbers in this document refer to the "marked-up copy" version of the manuscript.

Cordially,

Camila,

On behaf of my co-authors

| **EDITORIAL COMMENTS** | **ANSWERS** |
| --- | --- |
| Sincere apologies for the delay in getting back to you with a decision, which was due to challenges in securing the  necessary Reviewers. Many thanks for submitting your manuscript "Effect of a large cash transfer on mortality among  interpersonal violence victims: a cohort study" (PMEDICINE-D-25-01851R1) to PLOS Medicine. The paper has been  reviewed by subject experts and a statistician; their comments are included below and can also be accessed here:  ********  As you will see, the reviewers find the work of considerable interest but raise some very important concerns that would  need to be fully addressed in a revised manuscript. After discussing the paper with the editorial team and an academic  editor with relevant expertise, I'm pleased to invite you to revise the paper in response to the reviewers' comments.  However, it seems to us to be essential that the methodological concerns raised by Reviewer 1 are resolved in full. We  plan to send the revised paper to some or all of the original reviewers, and we cannot provide any guarantees at this  stage regarding publication. | Thank you. We appreciate it very much and have addressed all the points raised by the Reviewers. |
| In addition to these revisions, you may need to complete some formatting changes, which you will receive in a follow up email. A member of our team will be in touch with a set of requests shortly. If you do not receive a separate email within a few days, please assume that checks have been completed, and no additional changes are required. | We haven’t receive the separate yes; however, we remain available should any future adjustments be needed. |
| When you upload your revision, please include a point-by-point response that addresses all of the reviewer and editorial points, indicating the changes made in the manuscript and either an excerpt of the revised text or the location (eg: page and line number) where each change can be found. Please also be sure to check the general editorial comments at the end of this letter and include these in your point-by-point response. When you resubmit your paper, please include a clean version of the paper as the main article file and a version with changes tracked as a marked-up manuscript. It may also be helpful to check the guidelines for revised papers at http://journals.plos.org/plosmedicine/s/revising-your-manuscript for any that apply to your paper. | We organized the responses point by point and also submitted both the clean version and the marked version, as instructed. |

| **Comments from the reviewers:**  **# REVIEWER 1 (Statistician)** | **ANSWERS** |
| --- | --- |
| In all, a very interesting manuscript that addresses a relevant public health concern. However, there are  some important methodological concerns that preclude us from accepting the manuscript as is. As for some minor  comments, we suggest the authors avoid the use of the word "effect" in the title, which may erroneously suggest a  causal relationship despite having only these observational data (there was IPTW used, but no causal justifications or assertions were justified in the manuscript itself) | Thank you for your suggestion. We change the title for “Conditional cash transfer and mortality among interpersonal violence victims: a cohort study”. (lines 1-2, page 01) |
| As well, results from a Fine-Gray model are presented, but results don't seem to be commented on much (e.g., interpretations of subdistribution hazard ratios, etc.). | Thank you for your comment, we have now included a more detailed description of this analysis in both the Method and the Results sections .  Methods: “To address the potential bias of overestimating the risk of the event of interest due to competing risks in traditional Cox regression, we employed competing risk models using the Fine-Gray subdistribution hazard model.” (line 346, page 13)  Results: “Using the Fine–Gray subdistribution hazard model, the subdistribution hazard ratio for natural causes was 0.34 (95% CI: 0.28–0.41, p<0.001), while that for unnatural causes of death was 0.90 (95% CI: 0.67–1.19, p=0.461) among the beneficiary group. Therefore, the BFP was associated with a 66% decrease in the rate of natural causes and a 10% decrease for unnatural causes (non-significant) among individuals who either experienced a competing risk or were still alive.” (lines 418- 423, page 16) |
| One strength of the manuscript is the completeness of the administrative records and their ability to be linked. On line 188 there is a minor typo with a missing "N=". I'm not quite sure how the numbers line up; there seems to be a slight discrepancy (67487 from SINAN + 11286 from SIH - 1174 duplicates results in 77599 as opposed to the 78038  reported). It also would have been helpful to identify the N=14856 of the 29075 individuals in the analysis cohort who received the BFP exposure earlier in the manuscript for reader clarity. | Thank you for your comment. We included the “N” before 67,487 (line 237, page 09).  Regarding the data discrepancy, we apologize as the information on duplicates was correct but placed in the wrong line. In fact, the 1,174 duplicate cases were removed later, as can be better seen in Figure 1. We have moved this information to the appropriate part of the text and added the information on the total number of beneficiaries as requested. We have now improved the writing to make this information clearer:  Participant section: “As individuals with multiple records of interpersonal violence may have a higher risk of mortality^4^, we removed duplicate entries (1,174 cases, 4%) to mitigate biases in the association measurement. Finally, we removed individuals who had inconsistent data on dates, such as death before CadÚnico registration or the same start and end date for receiving BFP (n= 74, <1% of the total sample). Therefore, the study included 29,075 participants, of whom 14,856 (51%) were BFP recipients (figure 1).” (lines 248-251, pages 9-10) |
| We have two major methodological/statistical comments regarding the manuscript. The first is the degree to which you may ascribe associations as "due to" BFP. Receipt of BFP is based on strict eligibility criteria, nearly all of which are associated with differential outcomes. For instance, the strict eligibility requires /for/ these low-earning individuals are virtually all associated with better outcomes - maintaining attendance, keeping up with vaccinations, and specifying a health and nutrition protocol (implying that they have at least some regular contact with the healthcare system). Thus, a more interesting comparison, though not necessarily possible to evaluate, would be between BFP recipients and those who qualify under BFP eligibility requirements, but who for some reason did /not/ receive the financial benefit. This is to say that it is difficult to believe conclusions as to the benefit of the financial handout itself when receipt of those incentives is tied to behaviors that are likely conducive to lower mortality. | Thank you for this insightful comment. We agree that this is an important point. The BFP was designed as a program that combines both the cash transfer and the conditionalities, with the explicit goal of addressing immediate poverty through the monetary benefit and intergenerational poverty through the required health and education behaviors. These two components are therefore inseparable within the program’s structure and cannot be evaluated independently using available data. Regarding the suggestion to compare beneficiaries with individuals who meet all eligibility criteria but did not receive the benefit, this is unfortunately not feasible in our setting. Due to the high targeting efficiency of the BFP, virtually all individuals who are eligible do in fact receive the benefit. As a result, there is no meaningful group of eligible non-recipients in our dataset to allow for such a comparison. We have revised the text to improve this explanation:  Exposure section: “Recipients of the BFP must meet specific conditionalities to maintain their benefits, including minimum school attendance, keeping up with vaccinations, and monitoring the growth of young children. Additionally, pregnant or breastfeeding women are required to follow a specified health and nutrition protocol^22^. The conditionalities are grounded in the notion that tying benefits to constructive behaviors can further improve families’ chances of breaking free from poverty through educational attainment or health improvements^22^.” (lines 274-282, pages 10,11)”  We highlighted that both groups were identified through the CadÚnico. This system includes individuals who meet the criteria for poverty or extreme poverty and who may apply for various social benefits such as social benefits for people with disabilities, housing for low-income families, BFP, among others^19^. BFP has more stringent eligibility standards and focuses on families that constitute a defined subset within CadÚnico^22^. Therefore, not all individuals meet the eligibility criteria for participation in the BFP.” (lines 287-293, page 11) |
| Statements such as "The PAF analyses showed that 53% of overall deaths (PAF= 53%, 95% CI 42.1% -330 64.2%) could be avoided by the presence of BFP among those victims of violence," while perhaps accurately applying the definition of PAF mathematically, are thus misleading sociologically/statistically. | Considering this valuable observation, we decided to remove the PAF analysis, as the results presented in the other analyses already address the importance of the BFP in the mortality of victims of interpersonal violence. |
| The propensity score matching and IPTW procedure was used to try and mitigate these sorts of issues, but their use was not entirely satisfactory. Naturally, one of the largest predictors of mortality is simply age. However, it appears that age was treated as a very coarse categorical variable; a 25 year old and a 59 year old are very different in terms of risk for death due to natural causes. The matching procedure does not appear to be able to account for this. | We appreciate this important observation regarding the influence of age on mortality. To address the reviewer’s concern about the broad age range within the 25–59-year category, we conducted an additional sensitivity analysis introducing a more granular age stratification (etable 8 in Supplement 1). The revised categories are: <10 years, 10–24 years, 25–29 years, 30–59 years, and >60 years. This new categorization reflects key life-course transitions, including the potential entry into the labor market during young adulthood (Halfon et al., 2018). We included the following explanation in the Methods section:  “Finally, to check the broad age range within the 25–59-year category of age, we conducted an additional sensitivity analysis introducing a more granular age stratification (eTable 9 in Supplement 1).” (lines 383-385, page 14)  The results of this sensitivity analysis were consistent with the main findings. By refining the age categories, we were able to better capture the heterogeneity in life-course stages (Halfon et al., 2018) that previously existed within the 25–59-year group. As noted by Ali et al. (2019), propensity score methods compare exposed and unexposed individuals within each stratum, allowing for improved balance in the probability of receiving Bolsa Família across age categories. Thus, the combination of the revised age stratification and the propensity score approach likely contributes to reducing potential age-related confounding in the estimated association between BFP and mortality. |
| Moreover, we're not even convinced that propensity-based methods are even the ideal in this context. Given that you have essentially complete data from a variety of administrative databases, why not simply treat the participants as one massive cohort for whom you have very good follow-up? | Thank you for your comment. We agree that propensity-based methods are not the only appropriate approach for these analyses. In addition to the propensity-score models, we conducted several sensitivity analyses, including another propensity score-based method, the Kernel Matching approach (KM) (Table 3), Poisson (eTable 3 in Supplement 1) and Cox proportional hazards models with time-varying covariates (eTable 6 in Supplement 1). We also followed your suggestion to treat the participants as one large cohort with robust follow-up. Accordingly, we ran crude Cox models without propensity adjustment; these results are presented in etable 6 in Supplement 1. |
| You would then be able to use time-varying survival methods to examing adjusted hazard of mortality - the main exposure would be (time-varying) BFP status, adjusting for (time-varying) characteristics such as the ones examined in the present study. Using exact age at study entry would also avoid issues such as the ones raised at the beginning of this paragraph. The use of time-varying methods (for instance, use of BFP exposure as a time-varying covariate) would also avoid potential issues with immortal time bias by having differential classification of patients based on BFP status (i.e., a patient only being eligible to be in the BFP group if they didn't die very shortly after their hospitalization; for these patients, they would serve to inflate the hazard of death among non-BFP patients, simply because these patients didn't live long enough to be "BFP eligible" in the first place). | We appreciate the opportunity to include the time-dependent Cox analysis. The results confirmed our findings. We included an explanation of this analysis in the Method and Result sections (below) and a table with the results in the Supplement 1 (eTable 5). Regarding age, we clarified that age is only available at the study baseline. Therefore, we included this aspect as a study limitation (line 558-560, page 21): “In addition, the covariates were available only at the baseline of the study, which may limit their contribution to the outcome, especially age^39^.”  Sensivity analysis section: “Fourth, we ran Cox proportional hazards models with time-varying covariates^38^ considering BFP as a time-varying covariate to account for possible differences in the duration of benefit receipt and immortal time bias^38^.” (lines 383-385, page 14)  Results section: “Robustness checks using time-varying and stabilized IPTW Cox models confirmed these findings, suggesting a lower risk of mortality among BFP beneficiaries (eTable4 in Supplement 1).” (lines 424-426, page 16) |

| **Comments from the reviewers:**  **# REVIEWER 2** | **ANSWERS** |
| --- | --- |
| Overall, this paper makes an important contribution to the literature on cash transfers and violence. It was compelling and well done. I have some minor comments below. | Thank you for your comment. We have made efforts to implement the recommended corrections. |
| It would be helpful to more detail on which types of violence are reported in SINAN as there are certain types that might be less likely to result in hospitalization. For example, less severe cases like psychological violence or types of violence that people fear having reported like sexual abuse. More discussion is needed about this and how this links to the generalizability of the findings. | Thank you for this insightful comment. Indeed, certain forms of violence may be underreported in the system due to stigma and the challenges faced by healthcare professionals in identifying less visible types of interpersonal violence, such as psychological violence. We added a paragraph in the Discussion section addressing this limitation. In addition, we sought to clarify in the Methods section which types of violence can be reported in the systems:  Method: “This system includes physical, sexual, psychological, psychological abuses, neglect and other relational contexts perpetrated by family members, intimate partners, friends or strangers. The data is registered by professionals working in health facilities, and it has improved its reporting over the years. Individuals hospitalized due to interpersonal violence were also identified through the Hospitalization Information System (SIH).” (lines 196-200, page 08)  Limitation: “Furthermore, our study only included individuals that seek healthcare services, which likely represent the most severe types of interpersonal violence, potentially resulting in classification bias. Additionally, less visible forms of violence, such as psychological violence, may be underreported due to stigma and the difficulty healthcare professionals face in identifying them^51^.” (lines 548-553, page 20) |
| The authors state that the study could only examine the short-term effects of cash transfers on mortality. Because of this, it is likely that this analysis is picking up certain types of mortality and it would be helpful to have some analysis to understand more about that is going on. In particular, looking at modification by age (are these participants older already) and looking at types of mortality including chronic disease outcomes versus other types of morality. | We conducted an additional analysis to evaluate age as a potential effect modifier. The inclusion of the interaction term did not result in an improved model fit. Model comparisons based on the Akaike Information Criterion (AIC) and the Bayesian Information Criterion (BIC) were performed to assess this effect, and the corresponding results are presented in eTable 8 (Supplement 1). Regarding the exploration of other outcomes, such as chronic diseases, this was not possible due to the small sample size for subtypes of natural causes of death. We added an explanation of this analysis in the Methods section:  Methods: “Fifth, to assess whether age modified the association between exposure and mortality^39^, we included an interaction term between age and exposure in the model. We then compared models with and without the interaction term using the Akaike Information Criterion (AIC) and the Bayesian Information Criterion (BIC). The evaluation followed parameters established in the literature, in which an AIC difference smaller than 2 indicates that inclusion of the interaction term does not substantially improve model fit^40^ (eTable 8 in Supplement 1).” (lines 378-383, page 14) |
| More discussion is needed about what is meant by "interpersonal violence." I imagine that the effects would be different when we are thinking about violence within a household by a partner or a parent if the cash is going to the household and the perpetrator might receive the money. In these cases, it might be less likely for the victim to benefit from the cash. More discussion is needed about household violence and the implications here as well as how that shows up in the findings. | We included a definition of interpersonal violence in the Introduction:  “ Interpersonal violence is a multifactorial public health problem encompassing a wide range of relational contexts and manifestations. It may involve family members, intimate partners, friends, acquaintances, or strangers, and includes various forms such as child maltreatment, youth violence, violence against women, and elder abuse^1^. Its consequences extend across multiple domains, including physical, psychological, financial, sexual, neglect-related, and other dimensions^2^.” (lines 86-91, page 04)  Addionally, we appreciate the important insight on the role of a partner or a parent if the cash is going to the household and the perpetrator might receive the money. We improved the discussion to consider the context of domestic violence and the role of the perpetrator, given that the primary aggressor is often a family member (Peterson et al., 2019). We added the following text to the discussion:  Discussion: “Another factor that may influence women's health outcomes is the continued presence of the perpetrator within the household. Evidence from previous studies indicates that CTPs can enhance women's autonomy and bargaining power^41^, primarily because, in general, women are prioritized in the receipt of the social benefit^22^. Income transfer may alter household power or control dynamics, leading to separation processes when women are exposed to domestic violence^50^. However, it is also possible that perpetrators may appropriate the financial resources provided, thereby sustaining the cycle of domestic violence^41^. This dynamic is reflected in evidence indicating that CTPs have not led to a reduction in femicide rates^50^, underscoring the need for complementary public policies specifically aimed at addressing perpetrator behavior and preventing severe forms of violence.” (lines 509-519, page 19) |
| Duplicates were excluded but it's important to account for multiple violence experiences as cumulative effects are important with violence experiences. Could you instead make an indicate of multiple experiences and look at the effects among those with 1, 2+ or 0 experiences to see if there are differences there. | Thank you so much for your suggestion, we agree that the analyses by multiple violence experiences is very important and would add information to the paper. However, unfortunately due to limited number of cases that experienced more than one violence case, it was not possible to perfom it. We have now added to the limitations: " A longer follow-up may be necessary to fully assess the impact of receiving BFP on mortality long-term, especially among individuals with repeated exposure to violent events" (lines 546-548, page 20) |
| How were the covariates of living conditions and isolation defined? | We improved the text to explain these covariates. Living conditions included water supply, waste, sanitation and construction materials of the household.  We considered “isolation” when there was a single-person living in the household.  Method: “The following covariates were considered to estimate the propensity score: sex, age, education level, race, location of residence (rural x urban), living conditions (which included water supply, waste, sanitation and construction materials), isolation (single person in the household), Brazilian regions of residence, and year of CadÚnico registration.” (lines 319-323, page 12) |
| More information is needed about the distribution in the length of time receiving a grant after violence exposure and how long it was provided for. Examining modification by these factors would also be important to understand targeting of programs and how soon after violence experiences cash should be provided | We included a time-dependent analysis in which the duration of BFP receipt was considered, rather than only the receipt status (eTable7 in Supplement 1). In this way, we were able to observe that the effect remained. Furthermore, the average duration of benefit receipt after the violent event was 1.71 years (SD=1.10), as indicated in line 402, page 15. |
| More information is needed about missingness and why there is no missing data for outcome or exposure. Is this because people with missing data are excluded or not captured here? Were methods like multiple imputation considered? | We had missing data only for the covariates, which are not mandatory fields for professionals responsible for registering individuals in CadÚnico, although they represent a small portion of the dataset. These data were excluded in the main model. There are no missing values for exposure and outcome, as these are mandatory fields in the CadÚnico and SIM systems. We are adding an additional table in the supplementary material, using “missing” as a category in the final model to demonstrate the robustness of the results (eTable 5 in Supplement 1). Moreover, the number of missing values was not sufficient to perform a data imputation process. |
| Line 97 typo "the," | This was a writing error, and we thank you for pointing it out. We have removed this word. |

| **Comments from the reviewers:**  **# REVIEWER 3** | **ANSWERS** |
| --- | --- |
| This study aims to identify the role of a conditional cash transfer programme in altering trajectories of all-cause, natural, and unnatural mortality among a large sample of Brazilians between 2011 and 2015. Overall, the paper stands to make a contribution to the literature and should be of interest to the journal for publication. However, as written, there are significant limitations that preclude publication in its current form. Generally, the Introduction and Discussion are thin, undercited, and under-explained while the Methods and Results require some additional editing and explanation. Overall, the paper should be edited for English language fluency and be better connected in terms of writing style. Specific comments are below. | Thank you for your comment. We revised the English language throughout the manuscript, enhanced the introduction and discussion by incorporating a more theoretical foundation, added further details to the Methods section, and refined the presentation of the results. A more detailed description of these modifications is provided in the responses to the comments below. |
| Abstract - Some editing is required to make the Abstract more clear. Specifically, "linked to 100 million Brazilian Cohort baseline from 2011 to 2015" does not make sense as written | We have revised the text to: “This cohort study was conducted using data from 100 Million Brazilian Cohort, which were linked with interpersonal violence registries (2011- to 2015).” (lines 38-40, page 02) |
| Introduction  Additional explanation of the exact operating definition of interpersonal violence is needed in the Introduction. It remains unclear how broad a definition the study is using. Does it include, for example, gang violence? | We included a definition of interpersonal violence in the introduction section (below). It may involve family members, intimate partners, friends, acquaintances, or strangers, and includes various forms such as child maltreatment, youth violence, violence against women, and elder abuse. Its consequences extend across multiple domains, including physical, psychological, financial, sexual, neglect-related, and other dimensions:  “Interpersonal violence is a multifactorial public health problem encompassing a wide range of relational contexts and manifestations. It may involve family members, intimate partners, friends, acquaintances, or strangers, and includes various forms such as child maltreatment, youth violence, violence against women, and elder abuse^1^. Its consequences extend across multiple domains, including physical, psychological, financial, sexual, neglect-related, and other dimensions^2^.” (lines 86-91, page 04) |
| There are several examples of editing needed for English language clarity- e.g., "the last one" instead of "the latter" | Done. |
| Additional details on the BFP are needed for those unfamiliar with the programme. Please provide a paragraph on how it operates as well as the extant literature on its utility in violence and trauma outcomes | We provided more detailed information regarding the BFP in the Introduction section:  Introduction: “CTPs such as Bolsa Família Program have been shown positive effects on the health of their beneficiaries^12^. This is one of the largest conditional cash transfer initiatives globally^12^. Since its implementation in 2004, it has played a significant role in reducing poverty and extreme poverty among Brazilian families^13^. Although originally designed as a social equity policy, evidence indicates that the BFP also yields positive impacts on health outcomes and mortality reduction^12^. These effects are thought to result not only from the income transfer itself but also from the program’s conditionalities^12^. By providing financial support to families, the program contributes to improved living conditions and fosters engagement with healthcare services and school attendance^12^.” (lines 131-139, page 05) |
| There is no theoretical foundation or theory of change noted- why do authors believe that BFP would alter the trajectory of mortality among victims of interpersonal violence? | The role of BFP in health and mortality has been studied, and its main mechanisms have been explored (Cavalcanti et al., 2025; Pescarini et al., 2022; Ramos et al., 2021). Regarding specific populations, such as individuals exposed to interpersonal violence, we believe that the role of BFP operates through secondary prevention (given that in our study we only evaluated people who received BFP after hospitalization/ notification due to interpersonal violence), by facilitating referral to health services, as well as through the well-documented improvement of living conditions reported in other studies (Cavalcanti et al., 2025; Silva, 2018). We included some hypotheses to explain the relationship between BFP participation and mortality among individuals who have experienced interpersonal violence:  Introduction: “The role of BFP in health and mortality has been studied, and its main mechanisms have been explored^12,17,18^. Regarding specific populations, such as individuals exposed to interpersonal violence, we believe that the role of BFP operates through secondary prevention, by facilitating referral to health services, as well as through the well-documented improvement of living conditions reported in other studies^12, 19^.” (lines 147-151, page 06)  “We hypothesized that the reduction in mortality may occur by improving access to health services and mitigating physiological stress responses to violence as well as facilitating connection to other social protection programs, promoting social inclusion, better living conditions, and improved physical and mental health^2, 5^.” (lines 153-156, page 06). |
| Methods  Please provide additional information on the 100 million Brazilian cohort- though it is cited, more information on these data are required for a critical reading of the paper. | We had included a more detailed explanation about the 100 Million Cohort in the Supplement, but we are repositioning this explanation into the main text:  Method: “The 100 million Brazilian Cohort baseline was developed to investigate social determinants and the impact of social programs and policies on various health contexts in Brazil^23^. This dataset is based on information from over 131 million individuals who were registered between 2001 and 2018 in CadÚnico, the primary system for applying for social benefits in Brazil which include the BFP^22^. It includes socio-economic and sociodemographic information from poorer Brazilian individuals and their families who apply for social programs such as BFP^23^. To qualify and register with CadÚnico, families must have a per capita income of up to half a minimum wage or a total family income of up to three minimum wages^23^.” (lines 177-185, page 07) |
| It should be noted that there is tension between the Introduction (which states that most people do not seek healthcare after violent episodes) and Methods (in which violence is measured via health records in SINAN and SIH. This likely biases the results significantly | We included as a study limitation that our data included only individuals that seek healthcare services, which likely represent the most severe types of interpersonal violence. Additionally, we attempted to clarify the definition of interpersonal violence in the Introduction and Method sections as reported above.  Discussion: “Furthermore, our study only included individuals that seek healthcare services, which likely represent the most severe types of interpersonal violence, potentially resulting in misclassification bias.” (lines 546-553, page 20) |
| The non-beneficiary group is not well-described in the Methods, which leaves considerable doubt as to the comparability of the beneficiary and non-beneficiary samples. For instance, were the non-beneficiaries labelled as such because they are not eligible for BFP (e.g., their income is too high)? If so, this introduces substantial confounding that is not adequately controlled for using the covariates described in lines 245-247 | We included a more detailed explanation of the beneficiary and non-beneficiary groups in the Method section:  “The 100 million Brazilian Cohort baseline was developed to investigate social determinants and the impact of social programs and policies on various health contexts in Brazil^23^. This dataset is based on information from over 131 million individuals who were registered between 2001 and 2018 in CadÚnico, the primary system for applying for social benefits in Brazil which include the BFP^22^. It includes socio-economic and sociodemographic information from poorer Brazilian individuals and their families who apply for social programs such as BFP^23^. To qualify and register with CadÚnico, families must have a per capita income of up to half a minimum wage or a total family income of up to three minimum wages^23^.” (lines 177-185, page 07)  “We highlighted that both groups were identified through the CadÚnico. This system includes individuals who meet the criteria for poverty or extreme poverty and who may apply for various social benefits such as social benefits for people with disabilities, housing for low-income families, BFP, among others^19^. BFP has more stringent eligibility standards and focuses on families that constitute a defined subset within CadÚnico^22^. Therefore, not all individuals meet the eligibility criteria for participation in the BFP.” (lines 287-293, page 11)  Considering the socioeconomic differences between groups, we included in the model covariates available in the system that are sensitive to socioeconomic conditions, such as: water supply, waste, sanitation and construction materials. The methodological decision to employ propensity score estimation was also intended to model the probability of receiving the BFP—i.e., to approximate eligibility or non-eligibility—based on observable baseline sociodemographic characteristics. This approach aimed to mitigate potential selection bias, given that eligibility could not be fully ascertained using only the income cutoff available in our dataset. We included this information as follows:  “By applying stabilized IPTW, we aimed to balance differences between groups^35^. Although this cohort contains complete data, beneficiaries and non-beneficiaries may still differ because eligibility for the BFP depends on income and additional socioeconomic criteria that may introduce imbalance^22^. The use of propensity score estimation allowed us to model the probability of receiving the BFP based on observable baseline sociodemographic characteristics, thereby reducing potential selection bias, especially given that eligibility could not be fully determined using income cutoff alone. This methodological approach is consistent with prior studies^14-18, 32, 33^.” (lines 307-315, page 12) |
| "Cox" should be capitalized on line 280 | Done. |
| Like 285 should read "to test whether our results are an artifact…" OR "to test if our results are an artifact" | The correct is “to test whether our results are an artifact". (line 363, page 14) We have improved this in the revised version of the text. |
| There needs to be an explanation of the covariates and how they are measured as well- otherwise readers have no idea what "good household conditions" mean when reading the Results | We have added an explanation of the covariates:  Method: “The following covariates were considered to estimate the propensity score: sex, age, education level, race, location of residence (rural x urban), living conditions (which included water supply, waste, sanitation and construction materials), isolation (single person in the household), Brazilian regions of residence, and year of CadÚnico registration.” (lines 319-323, page 12) |
| Results  Line 304 "in" not "at" | Done. |
| Line 311 should be "and in Southeast Brazil" | Done. |
| Line 312 should be "and were registered" | Done. |
| Did the authors choose to conduct sex-disaggregated analyses? It is likely that women experiencing intimate partner violence have different trajectories compared to men who experienced community violence due to the differing drivers of each | A sex-stratified analysis was included and presented in the supplementary material (eTable 2 in Supplement 1). The findings indicated that the BFP exerted more pronounced effects among women, particularly concerning all-cause and natural mortality. Among men, a significant effect was observed only for natural causes, and its magnitude was smaller compared to that observed in women. We included information about this analysis in the Methods, Results, and Discussion sections:  Methods: “A stratified analysis was also performed by sex, considering that gender differences^2,5^ may influence mortality risk (eTable2 in Supplement 1).” (line 348-350, page 13)  Results: “The stratified analysis by sex showed that the BFP reduced mortality mainly among women, including both all-cause (HR 0.42, 95% CI 0.34–0.52, p=<0.001) and natural causes (HR 0.61, 95% CI 0.46–0.80, p=0.001), whereas among men, the reduction was observed only for natural causes (HR 0.69, 95% CI 0.52–0.91, p=0.009), (eTable2 in Supplement 1).” (lines 428-432, page 16)  Discussion: “BFP was also associated with reduced mortality especially among females, a group at higher risk for interpersonal violence^48^. Violence can negatively affect women’s health, added to the effects of gender inequity, increasing their mortality risk^6^. Previous research has highlighted the beneficial effects of CTPs on women’s health, particularly in programs such as the BFP, which target pregnant and breastfeeding women^23, 49^. Evidence from LMICs indicates that cash transfers improve women’s health and increase their use of health services, particularly for maternal and child care^49^.” (lines 501-507, page 19) |
| Discussion  It is clear that someone different wrote the Discussion than the Methods and Results- please harmonize language, acronyms, and syntax across the paper. | We acknowledge that some writing inconsistencies may have occurred, as English is not our first language. However, the manuscript was developed collaboratively — the first author prepared the initial draft, and all co-authors contributed equally to the review and refinement of the text. Now we have also carefully revised the manuscript to ensure consistency in the use of acronyms and terminology throughout. |
| The authors do not explain the secondary outcome results showing that BFP significantly reduced deaths due to natural, but not unnatural, causes. This, to me, is the more interesting finding since it may suggest that those who take advantage of BFP may improve their health overall through the conditionalities of the programme, but they still likely live in a disproportionately violent area of an already violent country, meaning that BFP in and of itself does not protect from homicide, suicide, or accidental death due to gun, gang, or intimate partner violence. Additional context for these findings would be appreciated. | Thank you for your suggestion, it is indeed a very important point. We have added a paragraph:  Discussion: “Surprisingly, the BFP was not associated with non-natural causes of death. This finding might have happened because victims of interpersonal violence have an increased risk of dying from violent causes, given that recipients often reside in areas characterized by high rates of urban violence^6^, and that a set of systematic public actions, together with the cash transfer, may be required for this already vulnerable population^2^. The findings further indicate that beneficiaries may experience improvements in overall health through the conditionalities of the BFP; however, the program may be insufficient to prevent deaths from violent causes^2^.” (lines 493-500, page 18) |
| A limitation regarding the fact that violence showing up in administrative records is likely the most egregious violence is needed- the study likely has significant misclassification bias that should be noted | Very important point, we have now included this information in the limitation section:  “Furthermore, our study only included individuals that seek healthcare services, which likely represent the most severe types of interpersonal violence, potentially resulting in misclassification bias. Additionally, less visible forms of violence, such as psychological violence, may be underreported due to stigma and the difficulty healthcare professionals face in identifying them^51^.” (lines 548-553, page 20) |
| The authors do not engage with the debate around the conditional nature of BFP and whether they recommend CCTs or merely NCCTs- though the proposed mechanisms lend themselves to recommending cash transfer programmes with conditionalities so violence victims are better connected to health and social services. | As we have not investigated a non-conditional program, we can only make recommendations regarding conditional ones. Accordingly, we have improved the text by further highlighting the importance of recommending conditional cash transfer programs:  Discussion: “Therefore, governments should integrate cash transfers into efforts to prevent deaths among those vulnerabilized by interpersonal violence, especially conditional programs that improve access to health and social services. Conditional CTPs should be advisable for governments to implement than non-conditional programs, as they have the potential to constructive behaviors, mitigating adverse health outcomes associated with interpersonal violence.” (lines 580-588, pages 21,22) |

| **Comments from the reviewers:**  **# REVIEWER 4** | **ANSWERS** |
| --- | --- |
| This study has many strengths- a large, national dataset. Use of IPTW and competing risks are strengths of the analyses. I think the questions is certainly interesting- can CTs reduce mortality AFTER IPV history so not a primary prevention question but a secondary prevention question which is also important but also seems important to know if CTs reduce IPV- among those who get CT (exposure) does outcome (IPV) reduce? which is related but different question | We appreciate the comments regarding the strengths of our study. Indeed, it is relevant to investigate the reduction of interpersonal violence after receiving the BFP, but this question has already been extensively explored in the literature (Machado et al., 2024). Our contribution lies in examining the effects of the BFP on mortality in a already victimized population that has experienced interpersonal violence. |
| How did you deal with repeat IPV events over time? Is it correct they were removed per lines 192-193? One concern with removing those with repeated violence is that they are at higher risk of death but those in violent relationships are more likely to experience repeated acts of violence so understanding if the cash reduced future acts is important, although perhaps a separate question- seems they were only 4% of the sample which seems small. | We appreciate the reviewer’s comment. As noted in line 250 (page 09), we excluded individuals with repeated interpersonal violence events to improve comparability across participants; those with recurrent episodes have a substantially higher risk of violent mortality, which could introduce heterogeneity difficult to address within the scope of the current analysis. Moreover, individuals with repeated interpersonal violence episodes represented only about 4% of the sample, which made it statistically unfeasible to examine them as a separate subgroup with sufficient precision. |
| Introduction- seems that describing the difference in sex with IPV is key as women are much more likely to be victims globally. Causes of IPV and intervention effects may differ by sex. | Thank you for your comment. We would like to highlight that our study did not include IPV exclusively, but included several forms of interpersonal violence, such as child abuse, domestic violence, elder abuse, and youth violence. Our data do not allow us to identify especifically whether the violence experienced by the woman was perpetrated by an intimate partner. We have now clarified this in the Introduction and Method sections. However, given the potential differences between men and women (Sardinha et al., 2022), we have now included a sex-stratified analysis. The results indicated that the BFP reduced mortality particularly among women. We added the description of these findings in the Supplement (eTable 2 in Supplement 1) and further discussed these results. The additions to the manuscript are listed below:  Introduction: “ Interpersonal violence is a multifactorial public health problem encompassing a wide range of relational contexts and manifestations. It may involve family members, intimate partners, friends, acquaintances, or strangers, and includes various forms such as child maltreatment, youth violence, violence against women, and elder abuse^1^. Its consequences extend across multiple domains, including physical, psychological, financial, sexual, neglect-related, and other dimensions^2^.” (lines 86-91, page 04)  Method: “SINAN is the system for mandatory reporting of interpersonal violence since 2011^26, 27^. This system includes physical, sexual, psychological, psychological abuses, neglect and other relational contexts perpetrated by family members, intimate partners, friends or strangers^26^. The data is registered by professionals working in health facilities, and it has improved its reporting over the years.” (lines 195-200, page 08)  Methods: “A stratified analysis was also performed by sex, considering that gender differences^2,5^ may influence mortality risk (eTable2 in Supplement 1).” (line 348-350, page 13)  Results: “The stratified analysis by sex showed that the BFP reduced mortality mainly among women, including both all-cause (HR 0.42, 95% CI 0.34–0.52, p=<0.001) and natural causes (HR 0.61, 95% CI 0.46–0.80, p=0.001), whereas among men, the reduction was observed only for natural causes (HR 0.69, 95% CI 0.52–0.91, p=0.009), (eTable2 in Supplement 1).” (lines 428-432, page 16)  Discussion: “BFP was also associated with reduced mortality especially among females, a group at higher risk for interpersonal violence^48^. Violence can negatively affect women’s health, added to the effects of gender inequity, increasing their mortality risk^6^. Previous research has highlighted the beneficial effects of CTPs on women’s health, particularly in programs such as the BFP, which target pregnant and breastfeeding women^23, 49^. Evidence from LMICs indicates that cash transfers improve women’s health and increase their use of health services, particularly for maternal and child care^49^.” (lines 501-507, page 19) |
| Just to clarify in the results for lines 322-323- :" for unnatural causes of death (HR 0.90, 95% CI: 0.67 - 1.19, p=0.461)" - so no significant decrease in unnatural causes or death only natural? So does this potentially include death resulting from IPV- so if a homicide from an intimate partner would it be included in this death? So per line 361- 362 where CCT have been shown to reduce violence 'primarily due to violent causes" it seems in this analysis unnatural causes of death include violent causes but no significant effect is observed here? Is this correct? Why do the authors think this is? | Indeed, the BFP had no effect on unnatural causes of death. We have added an explanation about this:  Discussion: “Surprisingly, the BFP was not associated with non-natural causes of death. This finding might have happened because victims of interpersonal violence have an increased risk of dying from violent causes, given that recipients often reside in areas characterized by high rates of urban violence^6^, and that a set of systematic public actions, together with the cash transfer, may be required for this already vulnerable population^2^. The findings further indicate that beneficiaries may experience improvements in overall health through the conditionalities of the BFP; however, the program may be insufficient to prevent deaths from violent causes^2^.” (lines 493-500, page 18) |
| In etable 2 (Poisson) why is the sample size so much smaller than in the cox model eTable 3? Effects are much larger in poisson model which makes me wonder if the samples truly are the same 'population' in the two models even with IPTW? | These tables refer to sensitivity analyses that were performed. The eTable 3 replicates the same analysis with the same population of individuals who experienced interpersonal violence (Table 3 of the main manuscript), however using a Poisson model to assess whether the results remain consistent under different model specifications.  The eTable 4, in turn, repeats the analyses for the entire population of victims of interpersonal violence, without excluding those who were registered in CadÚnico prior to the violent event. This analysis aimed to assess potential selection biases because of the exclusion of those registered in CadÚnico prior to the interpersonal violence. We explained this in the Method section:  Sensitivity analysis: “Third, to address potential selection bias, we conducted the analysis with the entire population victimized by interpersonal violence during the study period, including those registered in CadÚnico before the violent event (eTable 4 in Supplement 1).” (lines 367-369, page 14) |

References

Ali MS, Prieto-Alhambra D, Lopes LC, Ramos D, Bispo N, Ichihara MY, et al. Propensity Score Methods in Health Technology Assessment: Principles, Extended Applications, and Recent Advances. Front Pharmacol. 2019; 10: 1-19.

Cavalcanti DM, Ordoñez JA, da Silva AF, Basterra EL, Moncayo AL, Chivardi C, Hessel P, Sironi AP, Paes de Sousa R, Campello T, Souza LE, Rasella D. Health effects of the Brazilian Conditional Cash Transfer programme over 20 years and projections to 2030: a retrospective analysis and modelling study. Lancet Public Health. 2025 Jul;10(7):e548-e558.

Halfon N, Forrest CB, Lerner RM, Faustman EM (Ed.) Handbook of Life Course Health Development. Switzerland: Springer; 2018

Machado DB, Filha Siqueira NT, Castro-de-Araujo LFS, Alves FJO, Ramos D, Xavier EF et al. The relationship between cash-based interventions and violence: A systematic review and evidence map. Aggress Viol Beh. 2024; 75: 101909. https://www.sciencedirect.com/science/article/pii/S1359178923000964. (accessed Feb 15, 2025)

Pescarini JM, Campbell D, Amorim LD, Falcão IR, Ferreira AJF, Allik M, et al. Impact of Brazil's Bolsa Família Programme on cardiovascular and all-cause mortality: a natural experiment study using the 100 Million Brazilian Cohort. Int J Epidemiol. 2022; 51:1847-1861.

Peterson C, Liu Y, Merrick M, Basile KC, Simon TR. Lifetime Number of Perpetrators and Victim-Offender Relationship Status Per U.S. Victim of Intimate Partner, Sexual Violence, or Stalking. J Interpers Violence. 2021 Jul;36(13-14):NP7284-NP7297.

Ramos D, da Silva NB, Ichihara MY, Fiaccone RL, Almeida D, Sena S, Rebouças P, Júnior EPP, Paixão ES, Ali S, Rodrigues LC, Barreto ML. Conditional cash transfer program and child mortality: A cross-sectional analysis nested within the 100 Million Brazilian Cohort. PLoS Med. 2021 Sep 28;18(9):e1003509

Sardinha L, Maheu-Giroux M, Stöckl H, Meyer SR, García-Moreno C. Global, regional, and national prevalence estimates of physical or sexual, or both, intimate partner violence against women in 2018. Lancet. 2022 Feb 26;399(10327):803-813. doi: 10.1016/S0140-6736(21)02664-7.

Silva T.F. (Org). Bolsa Família 15 anos (2003 – 2018). Brasília: ENAP; 2018.
