# Supplementary material for: Conditional cash transfer and mortality among interpersonal violence victims: A cohort study
Source: PLoS Med. 2026 May 6;23(5):e1004673. doi: 10.1371/journal.pmed.1004673 (PMC13148827; doi:10.1371/journal.pmed.1004673)
Supplement: Attachment — Submitted filename: Point-by-point Plos Medicine 2nd-revision.docx [file pmed.1004673.s004.docx]

Dear Dr. Andreia Cunha,

Thank you for giving us the opportunity to resubmit a second revised version of our manuscript. We greatly appreciate the time and effort that you and the reviewers have put into providing valuable feedback on our work.

Below is a point-by-point response to the editors' and reviewers' comments and requests.

Please note that the line numbers in this document refer to the "marked-up copy" version of the manuscript.

Cordially,

Camila,

On behalf of my co-authors

| **EDITORIAL COMMENTS** | **ANSWERS** |
| --- | --- |
| Sincere apologies for the delay in getting back to you with a decision, which was due to challenges in securing all the necessary advice. Many thanks for submitting your manuscript "Conditional cash transfer and mortality among interpersonal violence victims: a cohort study" (PMEDICINE-D-25-01851R2) to PLOS Medicine. The paper has been reviewed by the original reviewers, including a statistician; their comments are included below and can also be accessed here: | Thank you. We appreciate it very much and have addressed all the points raised by the Reviewers. |
| As you will see, while two of the Reviewers are now happy with the revision, Reviewer 1 and Reviewer 3 maintain some important concerns that we must ask you to address in full. After discussing the paper with the editorial team and an academic editor with relevant expertise, I'm pleased to invite you to revise the paper in response to the reviewers' comments. Please include in the sensitivity analyses 10-year categories (i.e. 30-39, 40-49, 50-59) and remove any causal language from the Abstract and main text. We plan to send the revised paper to some or all of the original reviewers, and we cannot provide any guarantees at this stage regarding publication. | Thank you for the opportunity to revise our analysis, we really appreciate it.  We have now re-run all models using 10-year age intervals as a balancing covariate in the propensity score (IPTW) estimation, according to suggestion by the editor (eTable 9 in the Supplement). Overall, the results were similar in terms of the direction and magnitude of the association. However, differences were observed in the precision for overall mortality: although the point estimates continued in the same direction, statistical significance was attenuated  The differences observed after re-specifying age into narrower 10-year categories are most likely attributable to reduced statistical stability arising from sparse event counts. Increasing the number of age categories introduces additional parameters into the propensity score model, which may reduce effective sample size and result in smaller cell counts within specific age groups, particularly given the distribution of events. This phenomenon is also well documented in survival analyses (Stuart, 2010).  When age is modeled using finer categorization, the number of events per category may become insufficient to support precise estimation. This can lead to wider confidence intervals and increased sensitivity of effect estimates to random variation.  Regardless, to ensure transparency and fully address the reviewer’s and editor requests, we have retained the analyses using 10-year age strata in the Supplement (eTable 9).  Furthermore, we have removed causal language from both the abstract and the manuscript, as requested. |
| In addition to these revisions, you may need to complete some formatting changes, which you will receive in a follow up email. A member of our team will be in touch with a set of requests shortly. If you do not receive a separate email within a few days, please assume that checks have been completed, and no additional changes are required. | We appreciate the opportunity and will make all the necessary formatting adjustments. |
| When you upload your revision, please include a point-by-point response that addresses all of the reviewer and editorial points, indicating the changes made in the manuscript and either an excerpt of the revised text or the location (eg: page and line number) where each change can be found. Please also be sure to check the general editorial comments at the end of this letter and include these in your point-by-point response. When you resubmit your paper, please include a clean version of the paper as the main article file and a version with changes tracked as a marked-up manuscript. It may also be helpful to check the guidelines for revised papers at <http://journals.plos.org/plosmedicine/s/revising-your-manuscript> for any that apply to your paper. | We have provided point-by-point responses and implemented the requested revisions. |
| We ask that you submit your revision by Mar 12 2026 11:59PM. However, if this deadline is not feasible, please contact me by email, and we can discuss a suitable alternative. | Ok. |

| **Comments from the reviewers:**  **# REVIEWER 1** | **ANSWERS** |
| --- | --- |
| The authors have provided extensive additional analyses to address previous concerns. While much improved, there still remain statistical issues and inaccuracies. For instance, the revision to add "Therefore, the BFP was associated with a 66% decrease in the rate of natural causes..." regarding the Fine-Gray model is not correct; subdistribution hazards do not correspond to "rates" in that way (note also that epidemiologically, a rate must include a specified time interval in which the rate is calculated). | We appreciate the opportunity to revise the manuscript once again. We removed the term “rate,” which had been incorrectly used, and carefully reviewed the entire text to identify and correct any linguistic inaccuracies in the description of the results. |
| I am still not satisfied regarding the coarser age stratification wherein a 30 year old and a 59 year old are treated as having the same "age" for purposes of evaluating mortality in the study even though one is essentially double the age of the other (and has multiple times higher incidence rate ratio regarding leading causes of death such as cardiovascular disease). The new age stratification has essentially the same issue (30 year olds and 59 year olds being treated the same) as the previous analysis (25 year olds and 59 year olds being treated the same regarding age). | Thank you for your comment. We agree with your suggestion and, in line also with the editor’s request, we reran all models using 10-year age intervals. Please see our response to the editor’s second comment for further details. |
| Finally, although no comparator group is possible in the study, the concern regarding attribution of associations remains. To quote the previous review, "Receipt of BFP is based on strict eligibility criteria, nearly all of which are associated with differential outcomes. For instance ... maintaining attendance, keeping up with vaccinations, and specifying a health and nutrition protocol (implying that they have at least some regular contact with the healthcare system). ... This is to say that it is difficult to believe conclusions as to the benefit of the financial handout itself when receipt of those incentives is tied to behaviors that are likely conducive to lower mortality." More explicitly discussing this difficulty would make for a less misleading discussion. | We thank you for the opportunity to clarify this issue. We have addressed this aspect in the Discussion, as shown in the excerpt below:  “Similarly, it is difficult to disentangle the effect of receiving the benefit itself from the conditionalities attached to it. Therefore, it remains unclear whether the observed associations are attributable to the financial component per se or to behavioral changes required to maintain eligibility for the program.” (page 19, lines 524-528) |

| **Comments from the reviewers:**  **# REVIEWER 2** | **ANSWERS** |
| --- | --- |
| The authors have sufficiently addressed my comments. | Thank you. |

| **Comments from the reviewers:**  **# REVIEWER 3** | **ANSWERS** |
| --- | --- |
| The authors have adequately addressed my concerns. One note- the authors say on line 464 that "BFP was also associated with reduced mortality especially among females, a group at higher risk for interpersonal violence". This is not true- interpersonal violence writ large (which encompasses all forms of violent victimization- homicide, assault, etc.) affects far more men than women. However, women are much more likely to experience violence from an intimate partner, to experience sexual violence, and to be murdered by a romantic partner. The citation used here (Sardinha et al) does not address violent victimization among men and so is an inappropriate citation for the claim made. I suggest qualifying this statement by saying something about how BFP reduces mortality among females, likely by reducing financial strain within a family unit. | We thank you for the opportunity to revise this sentence. We have removed the sentence “…a group at higher risk for interpersonal violence” and added the sentence “…possibly by mitigating household-level financial stress^15^.” (page 17, lines 470-474). We have also removed the reference to Sardinha et al. |

| **Comments from the reviewers:**  **# REVIEWER 4** | **ANSWERS** |
| --- | --- |
| Overall, I think the authors have been very responsive to the comments of reviewers and this is an important analysis to publish. No further comments. | Thank you. |

| **General editorial requests:** | **ANSWERS** |
| --- | --- |
| We ask every co-author listed on the manuscript to fill in a contributing author statement, making sure to declare all competing interests. If any of the co-authors have not filled in the statement, we will remind them to do so when the paper is revised. If all statements are not completed in a timely fashion this could hold up the re-review process. If new competing interests are declared later in the revision process, this may also hold up the submission. Should there be a problem getting one of your co-authors to fill in a statement we will be in contact. Please do not add or remove authors without first discussing this with the handling editor. You can see our competing interests policy here: <http://journals.plos.org/plosmedicine/s/competing-interests>. | ok |
| Please upload any figures associated with your paper as individual TIF or EPS files with 300dpi resolution at resubmission; please read our figure guidelines for more information on our requirements: <http://journals.plos.org/plosmedicine/s/figures>. While revising your submission, we strongly recommend that you use PLOS's NAAS tool (<https://ngplosjournals.pagemajik.ai/artanalysis>) to test your figure files. NAAS can convert your figure files to the TIFF file type and meet basic requirements (such as print size, resolution), or provide you with a report on issues that do not meet our requirements and that NAAS cannot fix. | Done. |
| After uploading your figures to PLOS's NAAS tool - <https://ngplosjournals.pagemajik.ai/artanalysis>, NAAS will process the files provided and display the results in the "Uploaded Files" section of the page as the processing is complete. If the uploaded figures meet our requirements (or NAAS is able to fix the files to meet our requirements), the figure will be marked as "fixed" above. If NAAS is unable to fix the files, a red "failed" label will appear above. When NAAS has confirmed that the figure files meet our requirements, please download the file via the download option, and include these NAAS processed figure files when submitting your revised manuscript. | ok |
| Please ensure that the paper adheres to the PLOS Data Availability Policy (see <http://journals.plos.org/plosmedicine/s/data-availability)>, which requires that all data underlying the study's findings be provided in a repository or as Supporting Information. For data residing with a third party, authors are required to provide instructions with contact information (web or email address) for obtaining the data. Please note that a study author cannot be the contact person for the data. PLOS journals do not allow statements supported by "data not shown" or "unpublished results." For such statements, authors must provide supporting data or cite public sources that include it. | We have provided the information on data availability in the manuscript, as shown below:  “The information examined in this research is stored and managed by the Center for Data and Knowledge Integration for Health (CIDACS). Complete access to this data is limited due to its confidential nature and the specific licensing agreement that restricts its use to this study. Brazilian ethical guidelines, as determined by the Ethics Committee, prevent this data from being publicly shared. However, under certain conditions and if all ethical and legal criteria are satisfied, the data curation team at the institution may grant access to the data. For additional details, inquiries can be sent to [cidacs.curadoria@fiocruz.br](mailto:cidacs.curadoria@fiocruz.br). The code used in the analysis is available from Github [https://github.com/profacamilabonfim/Do-file-of-the-paper-Conditional-cash-transfer-and-mortality-among-interpersonal-violence-victims.git] and archived in Zenodo [10.5281/zenodo.18852494]" (page 22, lines 600-603) |
| We expect all researchers with submissions to PLOS in which author-generated code underpins the findings in the manuscript to make all author-generated code available without restrictions upon publication of the work. In cases where code is central to the manuscript, we may require the code to be made available as a condition of publication. Authors are responsible for ensuring that the code is reusable and well documented. Please make any custom code available, either as part of your data deposition or as a supplementary file. Please add a sentence to your data availability statement regarding any code used in the study, e.g. "The code used in the analysis is available from Github [URL] and archived in Zenodo [DOI link]" Please review our guidelines at <https://journals.plos.org/plosmedicine/s/materials-software-and-code-sharing> and ensure that your code is shared in a way that follows best practice and facilitates reproducibility and reuse. Because Github depositions can be readily changed or deleted, we encourage you to make a permanent DOI'd copy (e.g. in Zenodo) and provide the URL. | Ok. |
| FORMATTING - GENERAL *       Abstract: Please structure your abstract using the PLOS Medicine headings (Background, Methods and Findings, Conclusions). Please combine the Methods and Findings sections into one section. | Done. |
| At this stage, we ask that you include a short, non-technical Author Summary of your research to make findings accessible to a wide audience that includes both scientists and non-scientists. The Author Summary should immediately follow the Abstract in your revised manuscript. This text is subject to editorial change and should be distinct from the scientific abstract. Ideally each sub-heading should contain 2-3 single sentence, concise bullet points containing the most salient points from your study. In the final bullet point of 'What Do These Findings Mean?', please include the main limitations of the study in non-technical language. Please see our author guidelines for more information: <https://journals.plos.org/plosmedicine/s/revising-your-manuscript#loc-author-summary>. | Done. |
| Please express the main results with 95% CIs as well as p values. When reporting p values please report as p<0.001 and where higher as the exact p value p=0.002, for example. Throughout, suggest reporting statistical information as follows to improve clarity for the reader "22% (95% CI [13%,28%]; p</=)". Please be sure to define all numerical values at first use. | Done. |
| Please include page numbers and line numbers in the manuscript file. Use continuous line numbers (do not restart the numbering on each page). | Done. |
| Please cite the reference numbers in square brackets. Citations should precede punctuation. | Done. |
| FIGURES AND TABLES *       Please provide titles and legends for all figures and tables (including those in Supporting Information files). | Done. |
| Please define all abbreviations used in each figure/table (including those in Supporting Information files). | Done. |
| Please consider avoiding the use of red and green in order to make your figure more accessible to those with color blindness. | Not applicable. |
| SUPPLEMENTARY MATERIAL *       Please note that supplementary material will be posted as supplied by the authors. Therefore, please amend it according to the relevant comments outlined here. | ok |
| Please cite your Supporting Information as outlined here: <https://journals.plos.org/plosmedicine/s/supporting-information> | Ok. |
| REFERENCES *       PLOS uses the numbered citation (citation-sequence) method and first six authors, et al. | Done. |
| Please ensure that journal name abbreviations match those found in the National Center for Biotechnology Information (NCBI) databases (<http://www.ncbi.nlm.nih.gov/nlmcatalog/journals>), and are appropriately formatted and capitalised. | Done. |
| Where website addresses are cited, please include the complete URL and specify the date of access (e.g. [accessed: 12/06/2023]). | Done. |
| Please also see <https://journals.plos.org/plosmedicine/s/submission-guidelines#loc-references> for further details on reference formatting. | Done. |
| OBSERVATIONAL STUDIES  Abstract: Please include the study design, population and setting, number of participants, years during which the study took place (enrollment and follow up), length of follow up, and main outcome measures. | Done. |
| Please ensure that the study is reported according to the STROBE (or appropriate STOBE extension) guideline (available from: <https://www.equator-network.org/reporting-guidelines/strobe)> and include the completed STROBE (or STROBE extension) checklist as Supporting Information. Please add the following statement, or similar, to the Methods: "This study is reported as per the Strengthening the Reporting of Observational Studies in Epidemiology (STROBE) guideline (S1 Checklist)." When completing the checklist, please use section and paragraph numbers, rather than page numbers. | Done. |
| For all observational studies, in the manuscript text, please indicate: (1) the specific hypotheses you intended to test, (2) the analytical methods by which you planned to test them, (3) the analyses you actually performed, and (4) when reported analyses differ from those that were planned, transparent explanations for differences that affect the reliability of the study's results. If a reported analysis was performed based on an interesting but unanticipated pattern in the data, please be clear that the analysis was data driven. | Done. |
| Please state in the Methods section whether the study had a prospective protocol or analysis plan. If a prospective analysis plan (from your funding proposal, IRB or other ethics committee submission, study protocol, or other planning document written before analyzing the data) was used in designing the study, please include the relevant document(s) with your revised manuscript as a Supporting Information file to be published alongside your study and cite it in the Methods section. A legend for this file should be included at the end of your manuscript. If no such document exists, please make sure that the Methods section transparently describes when analyses were planned, and when/why any data-driven changes to analyses took place. Changes in the analysis, including those made in response to peer review comments, should be identified as such in the Methods section of the paper, with rationale. | Done. |
| **Journal Requirements:**  We have noticed that you have uploaded Supporting Information files, but you have not included a list of legends. Please add a full list of legends for your Supporting Information files after the references list. | Done. |

**Reference**

Stuart E. Matching methods for causal inference: A review and a look forward. Stat Sci. 2010; 25(1): 1-21. https://pmc.ncbi.nlm.nih.gov/articles/PMC2943670/
